# Vibration and Slope Conditions during Harvesting Affect Radish Mass Measurements for Yield Monitoring: An Experimental Study Using a Laboratory Test Bench

**DOI:** 10.3390/s23249744

**Published:** 2023-12-10

**Authors:** Shafik Kiraga, Md Nasim Reza, Milon Chowdhury, Md Ashraffuzzaman Gulandaz, Mohammod Ali, Md Sazzadul Kabir, Eliezel Habineza, Md Shaha Nur Kabir, Sun-Ok Chung

**Affiliations:** 1 Center for Precision and Automated Agricultural Systems, Irrigated Agriculture Research and Extension Center, Washington State University, Prosser, WA 99350, USA; shafik.kiraga@wsu.edu; 2 Department of Smart Agricultural Systems, Graduate School, Chungnam National University, Daejeon 34134, Republic of Korea; reza5575@cnu.ac.kr (M.N.R.); gulandazbari@o.cnu.ac.kr (M.A.G.); sazzadul.23@o.cnu.ac.kr (M.S.K.); eliezel.habineza@o.cnu.ac.kr (E.H.); 3 Department of Agricultural Machinery Engineering, Graduate School, Chungnam National University, Daejeon 34134, Republic of Korea; sdali77@o.cnu.ac.kr (M.A.); kabir1982@hstu.ac.bd (M.S.N.K.); 4 Agricultural Technical Institute, Division of Horticultural Technologies, Ohio State University, Wooster, OH 44691, USA; chowdhury.189@osu.edu; 5 Farm Machinery and Post-Harvest Processing Engineering Division, Bangladesh Agricultural Research Institute, Gazipur 1701, Bangladesh; 6 Department of Agricultural Industrial Engineering, Faculty of Engineering, Hajee Mohammad Danesh Science and Technology University, Dinajpur 5200, Bangladesh

**Keywords:** precision agriculture, radish, yield monitoring, impact plate, field slope, field vibration

## Abstract

Site-specific measurements of the crop yield during harvesting are essential for successfully implementing precision management techniques. This study aimed to estimate the mass of radish tubers using the impact principle under simulated vibration and sloped-field harvesting conditions with a laboratory test bench. These conditions included the conveyor speed (CS), impact plate layout (IP), falling height onto the impact plate (FH), the plate angle relative to the horizontal (PH), the field slope, and the vibration of the harvesting machine. Two layouts of impact-type sensors were fabricated and tested, one with a single load cell (SL) and the other with two load cells (DL). An adjustable slope platform and a vibration table equipped with vibration blades were utilized to simulate the slope and vibration effects, respectively. Calibrations were conducted to verify the accuracy of the sensor outputs, processed with the finite impulse response and moving average filters. Radish mass was estimated using an asymmetrically trimmed mean method. The relative percentage error (RE), standard error (SE), coefficient of determination (R²), and analysis of variance (ANOVA) were used to assess the impact plate performance. The results indicated that the SE for both impact plates was less than 4 g in the absence of vibration and slope conditions. The R^2^ for the single and double impact plates ranged from 0.58 to 0.89 and 0.69 to 0.81, respectively. The FH had no significant impact, while the PH significantly affected the mass measurements for both impact plates. On the other hand, the CS significantly affected the plate performance, except for the double-load-cell impact plate. Both vibration and slope affected the mass measurements, with RE values of 9.89% and 13.92%, respectively. The RE for filtered radish signals was reduced from 9.13% to 5.42%. The tests demonstrated the feasibility of utilizing the impact principle to assess the mass of radishes, opening up possibilities for the development of yield-monitoring systems for crops harvested in a similar manner.

## 1. Introduction

The radish (*Raphanus sativus* L.) is a globally significant vegetable, commonly eaten raw as a salad vegetable [1], which is also recognized for its potential medicinal properties [2]. As annual or biennial crops, radishes are typically grown in open fields. The global planting area and total annual production reached 3.1 million ha and 95.0 million tons in 2019, respectively [3]. However, despite their importance as a crop, their production has been declining worldwide [4]. For example, Japan experienced a 24% reduction in the yield of Daikon radish from 2006 to 2020 [5]. This decline can be attributed to various factors, including labor-intensive and time-consuming radish cultivation operations, limited mechanization, rising labor costs, and a diminishing workforce [6].

To address these challenges, various countries have begun promoting mechanization as a means to accelerate the harvesting and collection processes of radishes [7]. With the adoption of mechanization, there is an opportunity to enhance the management of radish fields by mapping the variability in yields and accounting for their spatiotemporal distribution in the field. Therefore, it is crucial to develop a yield-monitoring system for radish mass that is capable of instantly assessing yield and accounting for inter-field and intra-field variability. Real-time yield monitoring and spatiotemporal distribution analysis are critical components of precision agriculture, enabling farmers to verify current-season yields and providing guidance for future seasons [8]. Several commercial yield monitoring systems, including Green Star (Deere & Company, Moline, IL, USA), Advanced Farming Systems (AFS) (Case IH, CNH Industrial America LLC., Felton, DE, USA), and Grain-Trak (Micro-Trak System, Inc., Mankato, MN, USA) are currently available worldwide.

Several sensing components are employed for monitoring the yield of crops. These include grain flow sensors, moisture content sensors, and cutting width sensors [9]. The use of vision systems has also been tested recently [10]. Generally, the type of sensing system depends on the harvesting method and the harvesting machine structure [11]. For instance, contact-type mass flow methods that use impact-type sensors are mounted in the middle of grain transportation routes such as augers and grain tanks for monitoring grain yield [9]. These types of sensors measure and accumulate the weight of grains in the field. Although a similar sensing principle could apply to radish-harvesting machinery, the sensor design and installation location of these sensors need to be revised in accordance with the machine structure and nature of radish harvesting. Also, in contrast with grain harvesting where the mass flow rate is high, the mass of a single radish tuber is desired during harvesting. Therefore, modification in the design of the sensing components is desirable to suit the radish-harvesting method.

Impact-type sensors convert an impact force into a measurable electrical signal and have been extensively developed for grain crops. However limited examples exist for non-grain crops. Two impact-type weighing systems, one with four load cells and the other with a single load cell, were developed and tested during the 2004 and 2005 tomato harvesting seasons, and both impact plates were located at the end of the harvester conveyor boom [12]. These yield-monitoring systems worked well, with prediction errors of less than 2% under field conditions. Various load cell layouts have been used due to the irregular shapes of most crops and the varying structures of conveyor systems [11]. However, most of the earlier research has focused on validating the performance of commercially available yield-monitoring systems, with little emphasis on factors influencing sensor performance, such as machine structure and harvesting conditions. These factors greatly influence the choice and performance of a yield-monitoring system [13]. Therefore, it is crucial to develop accurate impact-type yield-monitoring systems designed with consideration for the specific harvesting characteristics of radishes and their growing conditions.

Harvesting conditions, such as slope and vibration, influence the quality and accuracy of yield-monitoring systems [14]. Additionally, factors that influence the mechanics of impact, including the coefficient of restitution, which represents the ratio of the velocity before and after impact, are critical crop characteristics [12]. These factors can affect the oscillatory response of load cells, potentially leading to a long settling time [15]. These effects are further increased in agricultural fields with uneven terrains and vibrations caused by various factors such as rotating shafts, transport operations like conveying, and other moving components in harvesting equipment [16]. Significant measurement errors have also been observed due to slope fluctuations, although limited studies have been conducted to assess this effect [17].

Test bench experiments enable researchers to systematically manipulate and study different variables, calibrate the system for accuracy, identify potential challenges, establish baseline data for field comparisons, ensure safety, and minimize unexpected issues during costly field trials. Prior to fabricating and conducting field tests, it is important to conduct experiments on a test bench to understand the impact of various harvesting conditions on the load cell signal output and obtain reliable yield data. Therefore, the objective of this study was to investigate the effects of different field operating conditions on radish mass measurements using the impact principle on a laboratory test bench. Specifically, this study aimed to (a) assess the impact of the conveyor speed, impact plate angle, falling height, and impact plate layout on radish mass measurements, (b) investigate the influence of slope and vibration levels on the loadcell signal, (c) and propose an appropriate signal processing and correction method. Section 2 presents the general structure and operating principles of radish-harvesting machinery (Section 2.1), the procedures taken to fabricate and set up the experimental test bench (Section 2.2 and Section 2.3), as well as the selection of experimental variables and levels (Section 2.4). The analytical techniques employed to evaluate radish mass estimations are described in Section 2.5. Section 3 details the results of experiments carried out without slope and vibration (Section 3.1), and the results for simulated vibration and slope conditions are described in Section 3.2.

## 2. Materials and Methods

### 2.1. Radish Harvesting Using Radish Collectors and Harvesters

**Figure 1:**
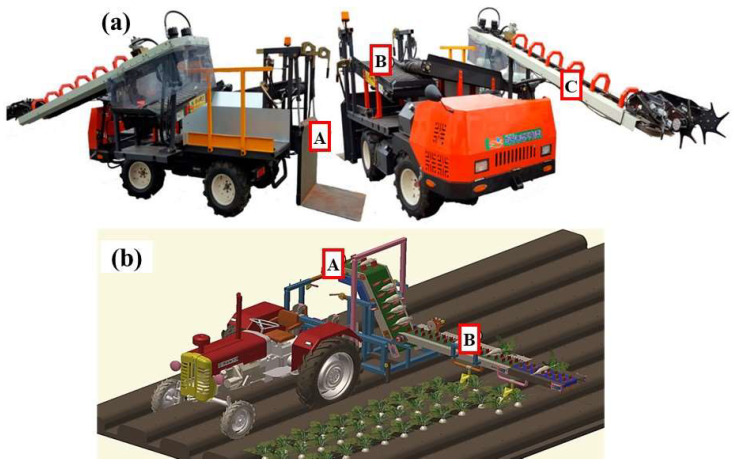
Self-propelled radish harvester (**a**), three-dimensional model of a tractor-mounted radish collector, and (**b**) impact plate installation position (A,A), load-out conveyor (B,B), and pull-out conveyor (C,B). Adapted from [18,20].

Radish harvesting is commonly performed using radish harvesters and collectors. Therefore, designing experiments on a laboratory test bench for radish yield estimation should take into account the harvesting conditions and the structural features of collectors/harvesters. Radish harvesters are usually equipped with a pull-out harvesting unit for holding the leaves, crushing the soil with a vibrating excavator blade, pulling radishes, and transporting them via the load-out conveyor, as shown in Figure 1a (Hope Farming Instrument Co., Ltd., Naju, Republic of Korea). The positioning guide installed on the transfer path guides the position of the crop during the transfer at a constant level, and the leaves are precisely cut at the desired position with a rotary cutting blade. The cut leaves are subsequently discharged, and the radish tubers are dropped onto the second conveyor. The second conveyor carries the radishes to the storing section, where they are collected in a bag and weighed using a weighing scale. The harvesting operation can be performed at speeds of up to 1.8 ms^−2^ [18]. However, considering the planting density of radishes (20 × 10 cm or 30 × 10 cm), lower speeds are preferred [19].

A three-point hitch is used to mount radish collectors onto tractors during the harvesting process, as shown in Figure 1b (Kukje Machinery Co., Ltd., Okcheon, Republic of Korea). The process of radish harvesting involves manually pulling the radish out of the soil and placing it on the first conveyor belt by hand. Subsequently, the stem is cut using stem cutters, after which the radish is transported to a storage bag positioned at the output end of the second conveyor belt. Harvesting is normally undertaken using a tractor with speeds ranging from 0.1 to 0.2 ms^−1^, which induces average vibration levels of approximately 0.41 ms^−2^ under loaded conditions in the second conveyor belt [18]. With both radish harvesting machinery types, the yield monitoring sensors are mounted at the end of the second conveyor (Figure 1aA and Figure 1bA) located at a vertical height of at least 200 mm and 830 mm from the ground for the radish collector and harvester, respectively [4,18]. The feed quantity to the storage containers is approximately 1 to 6 kgs^−1^ due to the planting pattern of radishes and the slow movement of radish harvesters during the harvesting process. In both scenarios, a significant advantage lies in the reduced presence of foreign matter on the second conveyor. This is achieved by cutting off and discharging the top radish leaves as they exit the first conveyor, for the radish harvester (Figure 1aC) and radish collector (Figure 1bB). This process ensures that the second conveyor carries a cleaner load, enhancing the accuracy of radish yield monitors based on the impact principle. Importantly, this design feature enables these monitoring systems to maintain their accuracy irrespective of the specific harvesting machinery employed.

### 2.2. Design and Fabrication of the Experimental Laboratory Test Bench

**Figure 2:**
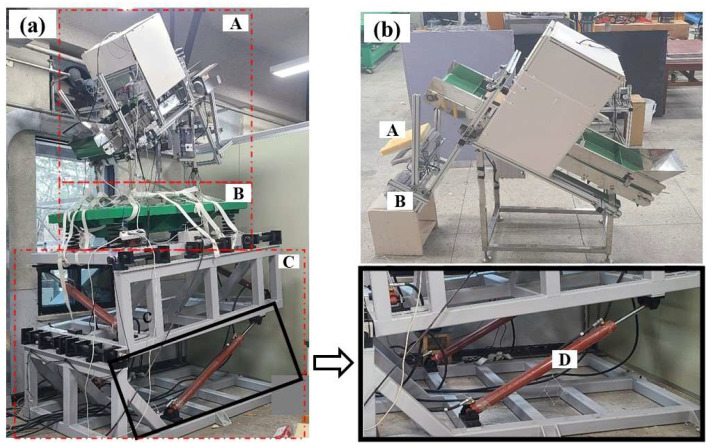
Yield-monitoring test bench for laboratory experiments: (**a**) conveyor (A), vibration table (B), slope platform (C), and slope gradient controller (D). Fabricated conveyor (**b**): impact plate (A) and data acquisition device (B).

The design and fabrication of the conveyor system for the laboratory tests followed the structure and operational conditions of the conveyor belt of radish collectors and harvesters. The conveyor output was chosen as most appropriate for the installation of an impact plate. A frame for holding the impact plate was constructed using aluminum profiles with dimensions of 40 mm × 40 mm (length (L) × width (W)). To test the effect of vibrations on sensor measurements, a vibration table (Figure 2aB) with sinusoidal oscillations under the shaking effect of a vibration motor was used. The oscillation frequency varied based on the applied rotational revolutions per minute (RPM) of the motor. To set different vibration levels, rotating blades were adjusted manually at each of the four end corners. Slope simulations were also performed using a sloped platform (Figure 2aC). Slope adjustments (both roll and pitch) were enabled via the attachment shown in Figure 2aD and actuated using a motor and a slope adjustment controller. In this experiment, the slope platform was operated under pitch orientations. During operation, the conveyor system was tightly fixed onto the vibration table and mounted onto the slope platform, as shown in Figure 2a. The specifications of the vibration table, the sloped platform, and the conveyor system are shown in Table 1.

### 2.3. Experimental Setup and Calibration Procedures for the Load Cells and Vibration Table

#### 2.3.1. Data Acquisition

The load cells (BCL-10L, CAS, Australia) provided outputs of 2.0 ± 0.2 mV V ^–1^ at a rated load of 100 N and were classified as a single-point type with a recommended measurement plate area of 300 × 300 mm. The plate size was chosen to accommodate the average geometrical dimensions of the radish tubers used in our experiments, which were 262 × 106 mm (length × width), respectively. The load cells exhibited a measurement range of up to 100 N with a measurement accuracy of ±0.2 N.

**Figure 3:**
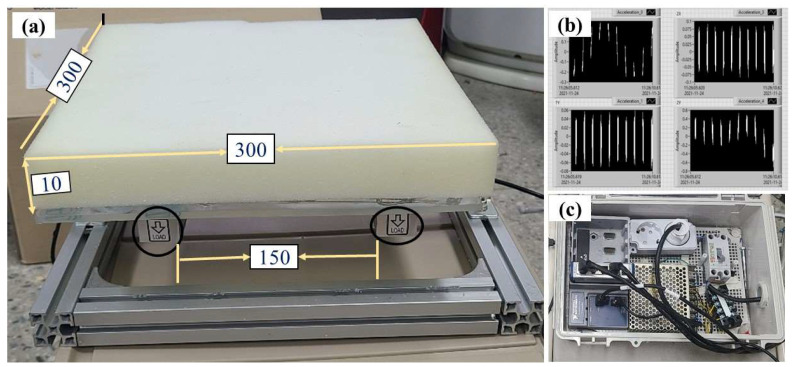
Data acquisition and load cell setup: double load cell impact plate (**a**), user panel programmed in a notebook (**b**), and data acquisition box (**c**). All dimensions are in mm.

A four-channel dynamic signal acquisition module (model: NI 9237; National Instruments, Austin, TX, USA) was used to collect the load cell signal in this study. This module was selected for its ability to conduct strain/load measurements with a zero inter-channel phase delay, which was especially suitable for the double load-cell impact plate configuration. A 10 V excitation input was provided to generate a 20 mV signal at a load of 100 N. The NI 9237 data acquisition module was placed into a four-slot DAQ-chassis (cDAQ NI-9174; National Instruments, Austin, TX, USA), and the output signals were transferred to the notebook via ethernet cables. A user panel was developed using a software program (LabVIEW 2020, National Instruments, Austin, TX, USA) for real-time data collection, visualization, and storage. Figure 3 illustrates the double impact plate and the data acquisition box used in the experiments. The impact plates were covered with a 3 mm and 10 mm thick acrylic plate and polyurethane foam, respectively, to protect the load cells from damage upon impact.

#### 2.3.2. Calibration of Load Cells

To test the suitability of the load cell sensors, the impact plate was adjusted into a horizontal position and loaded with five different weights one after another, with a measuring time of approximately 5 s at a sampling rate of 1 kHz. Each test weight was measured using a precision scale, where the minimum weight was 10 N and the maximum weight was approximately 90 N. The calibration was carried out before each experiment. Assuming that the mechanical loads on the impact plate cause linear differences in the sensor readings, and following the procedure proposed by [21] and the manufacturer’s specifications, the output of the data acquisition system was transformed into a theoretical force (N), as expressed in Equation (1).

(1) $$\mathrm{Force}\;\left(\mathrm{kN}\right)=\frac{Q}{R_{da}\times G_{lc}\times V_{in}}$$

where Q = the output of the data acquisition system (as a digital number), R*_da_* = the resolution for the data acquisition system (16,777,216 counts (24-bit)), G*_lc_* = the load cell gain (2.0 × 10^−3^ V V^−1^ (100 N)^−1^, and V_in_ = the load cell input voltage (10 V).

#### 2.3.3. Calibration of the Vibration Table

**Figure 4:**
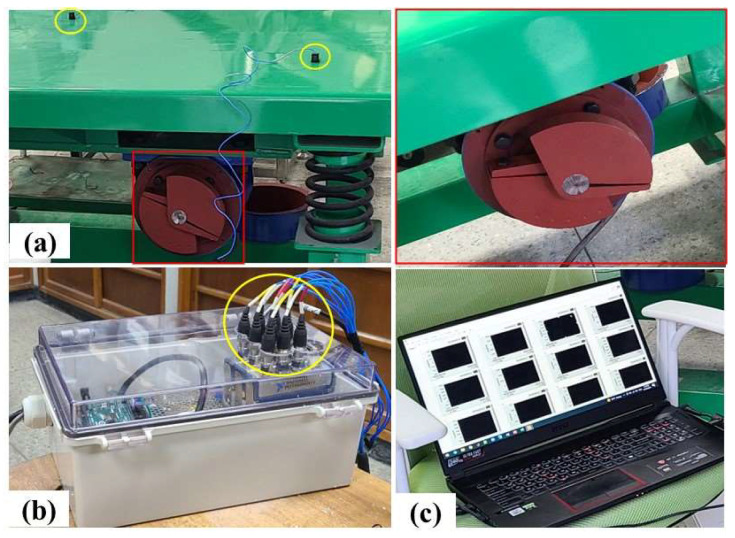
Calibration of the vibration table: placement of vibration sensors (**a**), data acquisition module (**b**), and a user panel programmed in a notebook (**c**).

Calibrating the vibration table was necessary to determine its frequency characteristics and guarantee its compliance with vibration levels encountered in real-field conditions. A series of tests were carried out by adjusting the relative positions of the vibration blades located at each of the four corners of the vibration table. In the calibration, a constant speed of 275 RPM was randomly chosen. For vibration measurements, three vibration sensors (model: 356A15, PCB Piezotronics Inc., Depew, NY, USA) were installed at different locations on the vibration table, as shown in Figure 4.

For the data collection, the sensors were interfaced with a data acquisition module (model: NI USB-6234, National Instruments, Austin, TX, USA) and a software program (LabVIEW 2020, National Instruments, Austin, TX, USA). A total of 32 calibration tests were carried out, with data collected at a sampling rate of 1 kHz. Vibration levels were measured in three different directions (X, Y, and Z). Equations (2) and (3) were used to calculate the average acceleration (*A_w_*) and the total acceleration (*A_v_*), respectively, as in [18].

(2) $$A_{W,axis}={\left[\overset{T}{\underset{0}{\int }}{\left[a_{W},\;axis\left(t\right)\right]}^{2}dt\right]}^{\frac{1}{2}}\;$$

(3) $$A_{v}=\sqrt{{\left(1.4\times A_{w,x}\right)}^{2}+{\left(1.4\times A_{w,y}\right)}^{2}+{\left(A_{w,z}\right)}^{2}\;}\;$$

### 2.4. Experimental Variables and Levels for Radish Mass Measurements

Two different layouts of impact plates were considered. One impact plate was supported with two load cells (Figure 3a) placed 150 mm apart, and the other was mounted with a single load cell placed in the middle of the acrylic plate. The experiments utilized the Jeju winter radish cultivar, which was purchased from an online market. This selection was made with careful consideration of the radish production and cultivation area [22]. Before each experiment, the radish mass and geometric properties were measured using a digital scale and a caliper, respectively. The experimental variables under consideration will be elaborated on in subsequent sections.

#### 2.4.1. Mass Measurement Tests without Slope and Vibration

The conveyor speed, falling height, and impact plate angle were the experimental variables investigated. The falling height was defined as the vertical height traveled by the radish sample to the impact plate. Height levels were selected in consideration of the orientation of radishes on conveyor belts during harvesting. Based on their geometrical structure, radishes are loaded radially, and to ensure the maximum impact force, they should impact the impact plate in the same orientation. In fact, varying mass estimates were reported for radial and axial impacts in experiments using cucumbers in [23]. Therefore, three levels of the falling height were selected (20, 30, and 40 cm). Vertical heights of more than 40 cm were not preferred to reduce the effect of drift in the radish trajectory to the impact plate.

The impact plate angle was the angle of the plate relative to the horizontal plane. Three different positions of the impact plate were considered (−10, −30, and −50°). Considering a maximum conveyor operating speed of 0.25 ms^−1^, three speeds were selected out of this range (0.05, 0.15, and 0.25 ms^−1^), whereas the horizontal distance between the conveyor and the impact plates was maintained as a constant.

#### 2.4.2. Mass Measurement Tests with Slope and Vibration

A series of experiments were performed considering three distinct conditions: only vibration, only slope, and both vibration and slope. The test bench was mounted onto the vibration table or the slope platform for the vibration or slope tests, respectively. For a combination of slope and vibration, the vibration table with the test bench was mounted onto the slope platform as shown in Figure 2a. A static test (pre-dynamic test) was carried out before each of the vibration or inclination tests to compare and determine the static characteristics before dynamic disturbances. Therefore, tests of either slope or vibration were described as dynamic tests. Nine experiments were carried out for each of the pre-dynamic and dynamic tests, resulting in eighteen experiments for each of the three conditions.

The maximum recommended slope for agricultural machinery is 26.79% (15°) [7]. Therefore, slope levels of 5.24% (3°), 10.51% (6°), and 15.84% (9°) were selected. Under loaded conditions, radish collector conveyor belts experience vibrations between 0.37 to 0.48 ms^−2^, and the levels are 1.01 to 1.66 ms^−2^ under unloaded conditions in radish fields [18]. The laboratory tests were carried out in consideration of the loaded conditions for vibration levels of up to 1 ms^−2^. Three vibration levels of 0.43, 0.78, and 0.98 ms^−2^ were selected. For a combination of slope and vibration conditions, experiments were performed based on the results obtained when these conditions were tested independently. Therefore, a vibration level of 0.43 ms^−2^ was selected and tested across slope levels of 3, 6, and 9°. All experiments were performed under three different speed levels of 0.05, 0.15, and 0.25 ms^−1^. A constant impact plate angle of −10° and a falling height of 40 cm were selected based on results from the tests without either slope or vibration. The experimental design is summarized in Table 2.

#### 2.4.3. Radish Signal Processing and Mass Estimation

**Figure 5:**
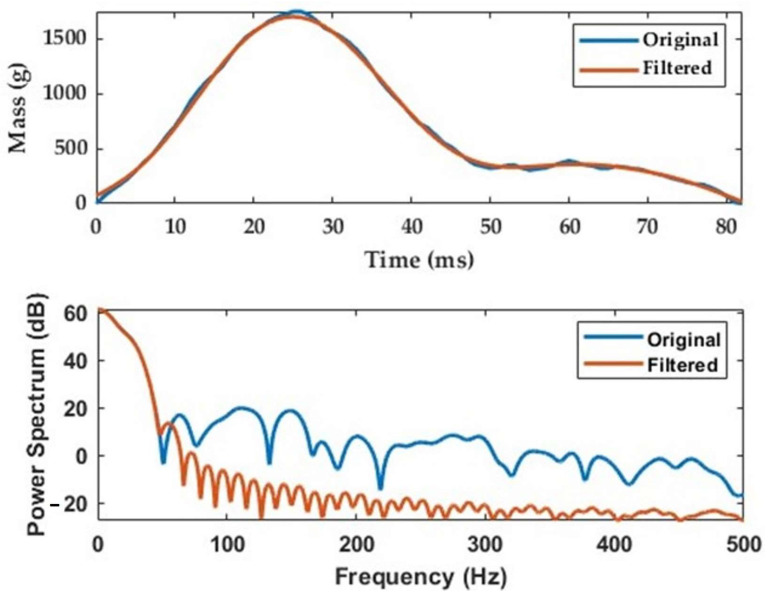
Results for the FIR filtering at a cutoff frequency of 50 Hz.

The load cell response to radish impacts (Figure 5) showed a rising signal upon impact, followed by a subsequent fall to the calibration state. Frequency analyses were conducted on the mass signal samples collected at 1 kHz. All frequency analyses were conducted using the discrete Fourier transform (DFT). The data were processed with a finite impulse response (FIR) digital low-pass filter with a cutoff frequency of 50 Hz. As a radish impacts the plate, the transient response should be returned to the initial state very quickly for subsequent measurements. Therefore, the FIR filter was adequate for radish mass measurements.

The filtered data from the load cell were further smoothed using the moving average approach to remove any significant peaks. Randomly selected averaging points of 5, 10, and 15 were used based on the total number of points for a radish sample signal impact of up to 80. The filtered output was used for radish mass estimations. As illustrated in Figure 5, the distribution of the load cell signal was heavy-tailed due to stochastic disturbances and short-time impacts. The signals were asymmetrical, which could lead to poor estimations of radish mass considering single-point readings from the load cell. Additionally, the transient response with lower extreme ends limited the use of the statistical mean, which is greatly affected by outliers that bias the resultant estimated mass. Therefore, a method for estimating radish mass using the asymmetrically trimmed mean was implemented, as described in [24]. First, the radish signal was sorted in ascending order, and then, 15% (p) and 5% (q) of the lowest and highest values were trimmed, respectively. This ensured the remaining 80% of the signal was free from extreme values. Trimming a smaller percentage of the upper values could enhance the capture of impact and significant events in the signal while still reducing the influence of potential outliers or measurement errors. On the other hand, trimming a larger percentage of the lower values could help to remove potential noise or low-impact observations. The final radish mass was calculated as shown in Equation (4).

(4) $$Trimmed\;mean\left(p,q\right)=\frac{\sum_{i\;=\;\lfloor pN\rfloor \;+\;1\;}^{\left\lfloor N-qN\right\rfloor }x_{\left(i\right)}}{N-\lfloor pN\rfloor \;-\;\lfloor qN\rfloor }$$

p ≥ 0, q ≥ 0, p + q < 1

where ⌊ ⌋ is the floor function, *N* is the total number of observations in the radish signal, ⌊*N*−*qN*⌋ is the upper bound index after trimming 5% of the highest values, ⌊*pN*⌋ + 1 is the lower bound index after trimming 15% of the lowest values, and *x_i_* is the individual mass measurement. 

**Figure 6:**
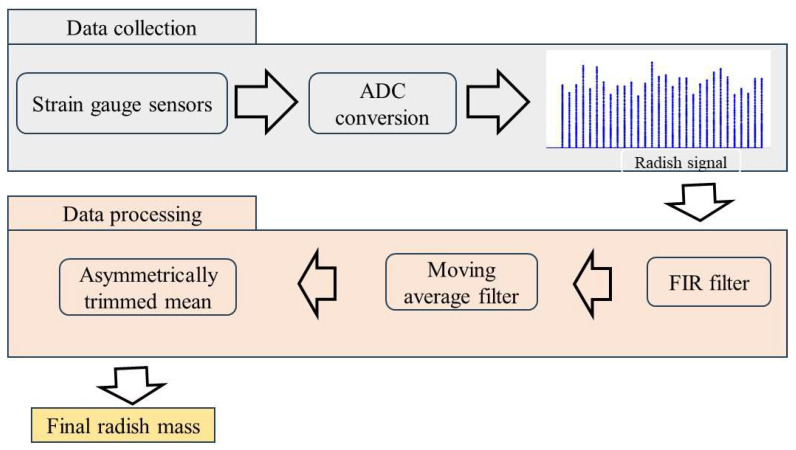
Structure of radish mass measurement and signal processing used in this study.

The developed radish mass measurement system is summarized in Figure 6. It consisted of strain gauge sensors for data acquisition, a signal-conditioning unit, and a mass estimation method.

### 2.5. Analytical Procedures

#### 2.5.1. Mathematical Modeling of the Radish Trajectory onto the Impact Plate

**Figure 7:**
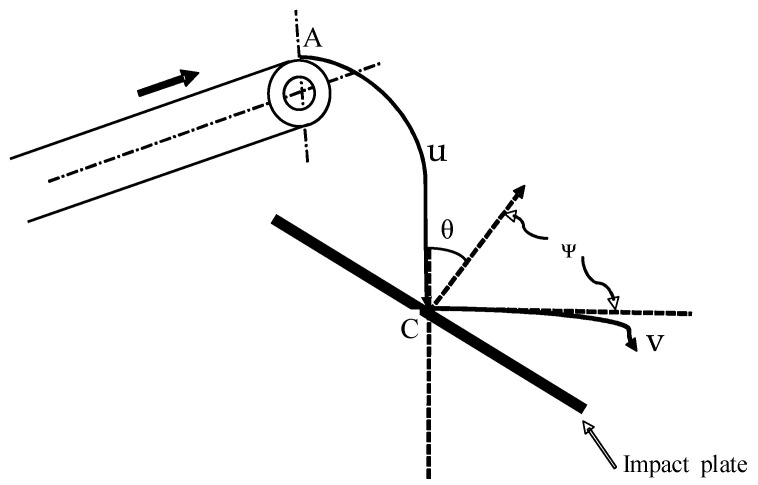
Effects of impact force on the impact plate.

Figure 7 illustrates the impact expected from the radish as it falls from the conveyor end onto the impact plate. From the principle of mechanics, the impact force (*F_p_*) is directly proportional to the mass (*m*) if the mass flow is under a constant speed difference, as shown in Equation (5) [12].

(5) $$F_{p}=\frac{m\;.\;\left(v-u\right)}{t_{2}-t_{1}}\;$$

where *F_p_* is the force of impact, *m* is the material mass, *u* is the initial speed, *v* is the velocity after impact, and *t* − *t*_1_ is the rate of momentum change.

If *θ* is the angle of approach and u is the speed at which the radish impacts the plate following path AC due to the gravitational force and the conveyor speed, the change in momentum in the normal direction via the impact plate can be mathematically expressed as in Equation (6) [12]. Substituting Equation (6) into Equation (5), the impact force (*F_p_*) can be mathematically expressed as in Equation (7).

(6) m(v.sinψ−u.cosθ) 

(7) $$F_{p}=\frac{m\left(v.sin\psi -u.cos\theta \right)}{△}\;$$

where *θ* is the incident angle, and *ψ* is the angle of reflection.

#### 2.5.2. Statistical Analysis of Load Cell Mass Measurements

The coefficient of determination (R^2^), percentage relative error (RE), and standard error (SE) were used to assess the experimental results. Analysis of variance (ANOVA) without interaction of factors was carried out using the above parameters as characteristic values, except R^2^. The experimental values were assessed for normality and variance uniformity using the Shapiro–Wilk test and Levene’s statistic, respectively, and transformed before analysis when necessary. The transformation functions used for the RE and SE values are shown in Equations (8) and (9), respectively.

(8) y=logx

(9) $$y=\frac{1}{x}$$

After the ANOVA tests, Duncan’s multiple range test at a 5% level of significance using SAS (SAS, Institute Inc, Campus Drive Cary, NC, USA) was conducted.

## 3. Results

### 3.1. Mass Measurements under Static Conditions

**Figure 8:**
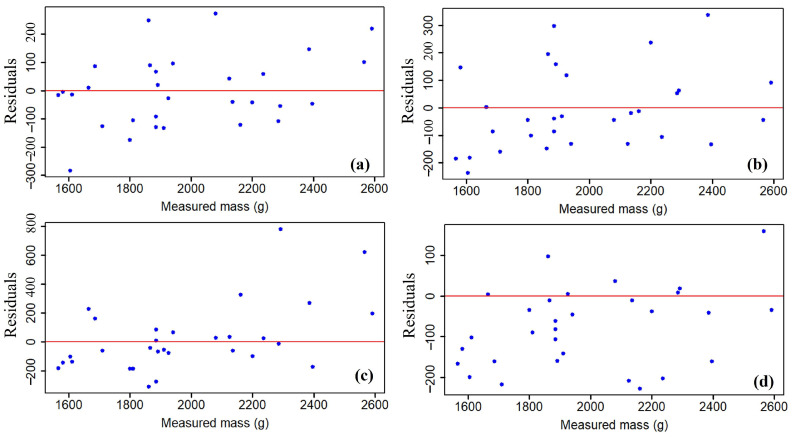
Residuals for estimated radish mass: single impact plate (**a**,**b**); double impact plate (**c**,**d**).

The residual plots in Figure 8 show the residuals plotted against the predictor variable for both the single and double impact plates. It can be observed that both impact plates were capable of measuring radish mass when tested under conditions without either slope or vibration. In the case of the single impact plate (Figure 8a,b), the errors were within −300 to 300 g, whereas for the double impact plate (Figure 8c,d), the errors were within −200 to 800 g. For instance, the impact plate appeared to overestimate radish mass in Figure 8c, while Figure 8d indicates residuals were more closely distributed around and close to the zero baseline. Generally, the single impact plate was more accurate than the double impact plate throughout the tests conducted. Table 3 shows the results from the ANOVA analysis, while Table 4 displays the overall statistical evaluation metrics of the two impact plates under static conditions.

For the single impact plate, the coefficient of determination during calibration and testing ranged from 0.94 to 0.98 and 0.58 to 0.89, respectively, whereas for the double impact plate, it ranged from 0.94 to 0.98 and 0.69 to 0.81, respectively. The high calibration values in both cases were expected since the mass sensor was given enough time to settle before readings were taken. The accuracy of each mass sensor layout during the tests could be further assessed by looking at the relative errors and standard errors. On average, the relative error for the single impact plate was 10.4%, while that for the double impact plate was 9.7%. The standard errors ranged from 2.29 to 2.76 g for the single impact plate and 2.3 to 3.4 g for the double impact plate, respectively. Generally, standard errors were less than 4 g in both cases. Considering the effect of the different factors on mass measurements, the tests revealed that there was a significant difference (*p* < 0.05) between the means of the measured mass across the different levels of the factors tested at a 5% level.

The height of the fall to the impact plate was not a significant factor for either impact plate, while the plate angle significantly affected mass measurements in both cases. On the other hand, the conveyor speed significantly affected the plate performance in contrast with the double-load-cell impact plate. Table 4 also reveals that as each of the factors increased, the errors in measurements tended to increase regardless of the impact plate layout tested. The changes in the radish falling height caused more variations in the measurements for the single impact plate, while changes in the plate angle for the double impact plate caused more variations. The mean relative errors in either case were 10.47% and 9.96%, respectively.

### 3.2. Radish Mass Estimation under Different Slope and Vibration Conditions

#### Results of Radish Signal Filtering

**Figure 9:**
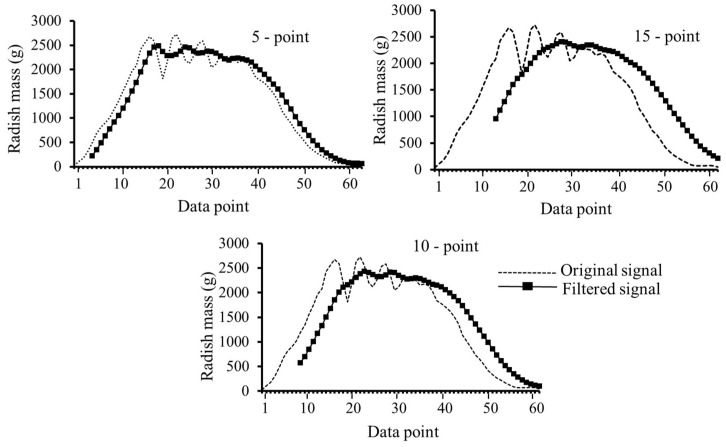
Filtered radish signals using different moving average points.

Figure 9 shows the effects of filtering and smoothing radish signals via the finite impulse response (FIR) and the moving averaging method, respectively. As expected, the signal peaks tended to flatten with increased averaging points. The resulting mass estimates when the filtered signals were further processed using an asymmetrically trimmed mean method for a combination of slope and vibration conditions are shown in Table 5. This indicated that increasing the averaging points increased the measurement errors. For instance, the 15-point moving average led to a mean relative error of 6.04% compared with the 5.42% for the 5-point moving average. Table 5 also compares results for the combination of vibration and slope tests (dynamic tests) and tests taken immediately before them (referred to as pre-dynamic tests). Measurements under the pre-dynamic conditions induced mean relative errors of 6.32%, smaller than the 9.13% calculated for the dynamic tests. Due to averaging using a 5-point filter and subsequent filtering, the mean relative errors were reduced by 41% from 9.13% to 5.42%, which was higher than the 34% obtained for the 15-point averaging.

As expected, large variations occurred at the highest conveyor speeds for each slope level. The relative errors at 0.25 m/s for 3°, 6°, and 9° were 13.74, 12.31, and 13.34%, respectively. The increase in the errors did not follow any trend. Table 5 indicates that the errors increased as the conveyor speed increased at 3° and 9°, whereas such a trend was not observed at 6°. Overall, the mean relative errors were considered low (<7%) for static tests and filtered radish signals, indicating the potential of dealing with variations in field conditions for accurate yield estimations.

ANOVA analysis revealed that the mean estimated mass across the different slope levels was significantly different (*p* < 0.0001). The mean mass estimate at the 9° slope level was higher and different from those at 3° and 6°. The mean estimates at 0.43 m/s^2^ and 0.78 m/s^2^ were significantly different (*p* < 0.0001) from those at 0.98 m/s^2^. The mean difference between the original and estimated mass were 110, 128, and 116 g for 0.43, 0.78, and 0.98 m/s^2^, respectively, indicating that radish mass estimates at 0.43 m/s^2^ were closer to the original measured mass than at other vibration levels. Mean mass estimates after subsequent radish signal conditioning for a combination of slope and vibration combinations revealed no significant differences (*p* = 0.930) (Table 6). This indicates the effectiveness of the radish signal smoothing, filtering, and trimming method used for estimating the mass.

**Figure 10:**
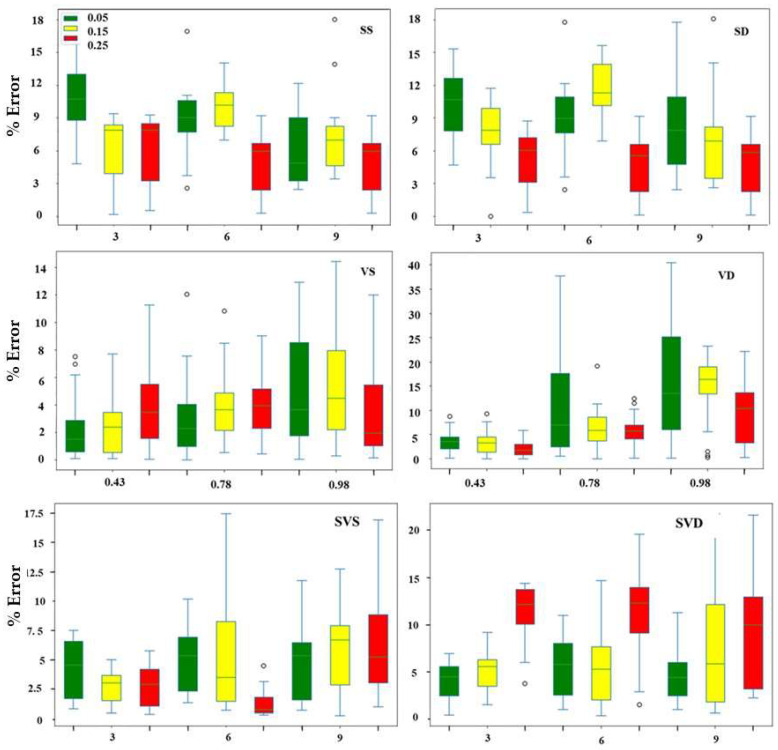
Percentage change in radish mass measurements under different slope and vibration levels.

Results for tests performed when the effects of slope and vibration conditions were tested independently are shown as box plots in Figure 10. For experiments carried out when the test bench was subjected to varying slope levels under static conditions (SS), the errors were almost uniform across the different slope levels. The errors tended to decrease from 3° to 9° from the minimum (0.05 m/s) to the maximum (0.25 m/s) speed, in contrast with 0.15 m/s, where maximum errors were observed at 6°. A relatively similar trend was observed under dynamic tests with minimum percentage errors observed at 9°. The mean relative errors calculated from these experiments indicated that SS (7.62%) was more precise than SD (13.92%), corresponding to a 45.3% absolute percentage difference. When the test bench was subjected to vibration conditions only, the percentage errors increased with the increase in the conveyor speed for the static (VS) and dynamic (VD) conditions. However, this trend was more significant under dynamic conditions. The mean relative errors for the VD and VS calculated from these experiments were 9.89% and 5.22%, respectively, giving an absolute percentage difference of 47.2%. This indicated that vibrations induced more errors than slope conditions (45.3%).

For a combination of slope and vibrations under dynamic conditions (SVD), the minimum percentage errors were observed at a slope of 3° and the maximum at a slope of 9°. However, no clear trend of the effect of speed on these measurements was observed, although maximum errors were observed at the highest speed. Across all speeds, the errors observed under SVD were higher than those under SVS, which proved that slope and vibration induced errors in the measurements, which could not be removed with smoothing or filtering. This means there could be multiple sources of errors in the radish signals. From Figure 10, it appears that the behavior of the test bench varied based on whether it was subjected to slope, vibration, or a combination of these conditions, which influenced the mass estimations. Each of these conditions influenced measurements in different ways, proving the necessity of investigating potential sources of error.

## 4. Discussion

### 4.1. Mass Measurement Tests without Slope and Vibration

The falling height from the conveyor output to the impact plate was not a significant factor for radish mass measurement for either impact plate, indicating that radishes hit the impact plates with the same speed and maintained the direction of the discharge trajectory from the conveyor belt. In such cases, a direct relationship between the impact force and mass flow exists [25]. Under practical conditions, this would be a desirable effect as it would lead to consistent mass measurements. The relative errors across different heights were consistently below 12% but slightly higher than those observed for grain yield in [26]. The difference could be due to the differing harvesting methods. The plate angle significantly affected mass measurements for both impact plates. According to Equation (7), the difference between the velocity of separation and approach is directly proportional to the impact force. At higher plate angles, the angle of approach reduces, which increases its cosine. Therefore, it is possible that for such elastic collisions between a radish and the impact plate, the velocity difference depends upon the energy loss upon impact, which is expected to vary at different positions of the plate. This was more noticeable for double impact plates, where the relative errors increased as the plate angle increased. This may have been due to the large cosine value of the angle of approach, and the reduced time of impact. No double impacts were observed in the experiments, in contrast with [27], which reported that angles less than 37° could lead to this effect. The difference in the observations could be due to the differences in the shape and size of radishes tested in this research and onions, which were tested in that study. In fact, varying sizes and shapes of specialty crops can influence the output of the designed yield monitors [28]. Considering the conveyor speed, the measured mass was significantly affected for the single impact plate but not for the double-load-cell impact plate. For irregularly shaped objects such as radishes, the trajectory during free fall could have deviated more significantly as the falling speed increased. Therefore, it is possible that at higher speeds, the orientation of the radish slightly changed as it exited the conveyor output, affecting the resultant impact. Findings in [23] agreed with our results, although they experimented at a single angle, considering different object orientations, and they suggested that the resultant impact force is dependent on the drop orientation.

### 4.2. Mass Measurement Tests with Slope and Vibration

Vibration and slope affected mass measurements, as found in [29], but the impact plate was more precise under vibration than slope conditions, indicating that good measurements can be achieved under field conditions with minimal fluctuating landscapes. In fact, ANOVA analysis results revealed significant differences (*p* < 0.05) in the mean mass estimates across the different slope levels, with a slope of 9° producing values that highly deviated from the original mass measurements compared with 3° and 6°. It is possible that the force with which the radish impacted the plates was reduced as the slope level was increased. Mass measurements under the different vibration levels were significantly different (*p* < 0.05), with optimal estimates at 0.43 m/s^2^, and the relative errors increased with the increase in vibration levels. This could be attributed to (1) the change in orientation as the radish hit the impact plate, or (2) the high settling time of the load cell sensors [30]. This is in agreement with [23], who found that axial impacts produced higher impact force than radial impacts for an object dropped from the same height. Furthermore, disruptions in the radish trajectory could lead to impurely vertical heights, which would reduce the force of impact as the radish hits the sides of the impact plate, a phenomenon observed at higher vibration levels. It was concluded that eliminating possible drift in the trajectory path was the key to improving load-cell-based mass measurements [31]. In this case, blocking radish deviations to the limited space of the impact plate would be desirable. Although positioning the impact plate at a height close to the conveyor output would reduce the errors, most conveyor belts are designed with flaps which limit the minimal height placement of the impact plates. Errors due to vibration could also result due to complex signals normally experienced under field conditions [32]. In such cases, the energy method was recommended by [12], which relates the measured mass to the signal content. However, for the individual-based weight measurements used in this study, the load cell signals were not complex enough to require that approach. It was observed (Figure 10) that increasing the conveyor speed reduced the effects of slope on mass measurements. Errors due to slope variations could be attributed to the gravitational acceleration of the mass flow sensor [33].

## 5. Summary and Conclusions

This study aimed to estimate the mass of radish tubers using the impact principle under simulated harvesting conditions on a laboratory test bench that included both slope and vibration effects. Based on the objectives of this paper, the following conclusions were obtained:

- Standard errors (<4 g) can be achieved for impact-based radish mass estimations for tests conducted without slope or vibration effects on a laboratory test bench.
- The machine structure and orientation of the impact plate, as well as its layout, had a significant effect on radish mass measurements. In contrast, the harvesting method, represented by the falling height of the radish to the impact plate, had no significant effect on the measurements.
- Uneven field conditions represented by the various slope and vibration levels significantly influenced radish mass estimates. For each slope level, maximum errors were observed at the highest speed tested (0.25 m/s). These were 13.74, 12.31, and 13.34% at 3°, 6°, and 9°, respectively. Tests under vibration produced a mean relative error of 9.89%, lower than that under various slope conditions (13.92%). Tests combining both slope and vibration induced a relative error of 9.13%. This was reduced to 5.42% via subsequent signal filtering.
- Based on the harvesting conditions tested in this study, a single impact plate placed at an angle of −10° and a radish falling height of 40 cm are recommended as suitable for radish measurements under conveyor speeds of less than 0.1 m/s.

It should be noted that the evaluations completed in this study were conducted under laboratory conditions, permitting precise control of radish flow, vibration, and slope levels. Variations from the results reported herein would be expected for complete harvesters operating under field conditions. Future work should focus on testing different impact plate layouts, as the distribution of force on the impact plate upon radish impact requires more analytical study. Furthermore, the implementation of slope and vibration sensors and signal filtering to account for slope and vibration in radish mass measurements will minimize the slope and vibration effects noted in this study. For signal filtering, it should be noted that the signal-processing techniques employed in individual mass-based sensing, as used in this study, may be different from those used when the whole products in the harvesting machine are measured. The yield-measuring system developed in this study and the subsequent analysis can provide a reference for developing yield-monitoring systems for other crops that are harvested in similar ways.

## Figures and Tables

**Table 1:** Overall specifications of the laboratory test bench setup.

Item		Specification		
Vibration table	Weight (kg) (kg)	Power rating (kW)	RPM	Dimensions (L × W × H) (mm)
	560	1.1	200 ~ 3600	2000 × 1000 × 900
Slope platform	Slope types	Measuring range (°)	Dimensions (L × W × H) (mm)	
	Pitch; roll	0~15	2200 × 1100 × 2000	
Conveyor system	Conveyor speed (m/s)	Angle of incline (°)	Dimensions (L × W × H) (mm)	
	0~0.25	27	1700 × 500	

**Table 2:** Experimental variables and levels for laboratory bench tests.

Experimental Variable			Levels		
**Mass measurement without slope and vibration**					
	**Conveyor speed (m** **s^–1^)**		0.05	0.15	0.25
	Falling height (cm)		20	30	40
	Impact plate angle (°)		−10	−30	−50
	Impact plate layout (no. of load cells)		Single	Double	
**Mass measurement with slope and vibration**					
**Measurement with vibration**		Conveyor speed (m s^−1^)	0.05	0.15	0.25
		Falling height (cm)	40		
		Impact plate angle (°)	−10		
		No. of load cells	Single		
		Vibration (m s^−2^)	0.43	0.78	0.98
**Measurement with slope**		Conveyor speed (m s^−1^)	0.05	0.15	0.25
		Falling height (cm)	40		
		No. of load cells	Single		
		Impact plate angle (°)	−10		
		Slope(°)	3	6	9
**Measurement with slope and vibration conditions**		Conveyor speed (m s^−1^)	0.05	0.15	0.25
		Falling height (cm)	40		
		Impact plate angle (°)	−10		
		No. of load cells	Single		
		Vibration (m s^−2^)	0.43		
		Slope (°)	3	6	9

**Table 3:** ANOVA results for investigating the significance of the effect of different factor levels on radish mass measurements.

Source	Sum of Squares	df	Mean Square	F-Value	Pr > F
Model	0.0224	6	0.0112	6.31	0.0029
Error	0.0238	20	0.001		
Total	0.0461	26			

**Table 4:** Averages for the statistical indicators for each experimental factor level.

Single-Load-Cell Impact Plate					
		Calibration (n = 5)	Validation (n = 30)		
	Level	R^2^	RE (%)	SE (g)	R^2^
Conveyor speed	0.05	0.97	8.6 ^a^	2.76 ^a^	0.89
	0.15	0.98	9.1 ^a^	2.64 ^a^	0.77
	0.25	0.96	13.6 ^b^	2.45 ^b^	0.75
Falling height	20	0.99	10.7 ^a^	2.51 ^a^	0.78
	30	0.95	8.7 ^a^	2.62 ^a^	0.85
	40	0.96	11.9 ^a^	2.72 ^a^	0.58
Plate angle	−10	0.97	7.5 ^a^	2.29 ^a^	0.84
	−30	0.94	8.2 ^a^	2.48 ^a^	0.82
	−50	0.96	15.7 ^b^	2.91 ^b^	0.69
**Double-Load-Cell Impact Plate**					
		**Calibration (n = 5)**	**Validation (n = 30)**		
	**Level**	**R^2^**	**RE (%)**	**SDE (g)**	**R^2^**
Conveyor speed	0.05	0.98	8.4 ^a^	2.7 ^a^	0.81
	0.15	0.97	9.5 ^a^	2.7 ^a^	0.77
	0.25	0.95	10.9 ^a^	2.7 ^a^	0.74
Falling height	20	0.96	10.5 ^a^	2.3 ^a^	0.75
	30	0.97	8.8 ^a^	2.9 ^a^	0.80
	40	0.99	9.6 ^a^	2.6 ^a^	0.76
Plate angle	−10	0.96	8.3 ^b^	2.3 ^b^	0.80
	−30	0.98	7.9 ^b^	2.5 ^b^	0.82
	−50	0.94	13.7 ^a^	3.4 ^a^	0.69

^a,b^ The different superscripts show the differences in averages within a factor of 5% level of significance.

**Table 5:** Combined slope and vibration effect on radish mass measurements.

Experimental Factor		Static Tests	Experimental Factor	Dynamic Tests	Moving Average		
Vibration Level (m/s^2^)	Conveyor Speed (m/s)	RE (%)	Slope (°)	RE (%)	N		
					5	10	15
0.43	0.05	6.61	3	4.52	3.6	3.4	6.2
	0.15	4.89		6.78	3.3	4.2	6.8
	0.25	5.10		13.74	4.8	5.8	4.7
	0.05	7.02	6	8.05	6.6	6.2	7.6
	0.15	9.29		7.68	7.4	5.7	6.1
	0.25	2.39		12.31	7.8	8.1	9.2
	0.05	6.46	9	7.2	4.7	7.4	3.8
	0.15	9.09		8.59	4.8	4.9	3.8
	0.25	6.49		13.34	5.7	5.5	6.2
Mean error, %		6.37		9.13	5.42	5.69	6.04

**Table 6:** ANOVA results for investigating the significance of the differences in mean mass estimates for a combination of slope and vibration levels.

Source	Sum of Squares	df	Mean Square	F-Value	Pr > F
Model	4616.667	2	2308.333	0.07	0.930
Error	2,594,188.889	78	33,258.832		
Total	2,598,805.556	80			

## Data Availability

All the data reported herein are available from the authors upon request.
